# Acteoside relieves mesangial cell injury by regulating Th22 cell chemotaxis and proliferation in IgA nephropathy

**DOI:** 10.1080/0886022X.2018.1450762

**Published:** 2018-04-30

**Authors:** Lu Gan, Xiaozhao Li, Mengyuan Zhu, Chen Chen, Huimin Luo, Qiaoling Zhou

**Affiliations:** a Department of Nephrology, First People’s Hospital of Yunnan Province, Kunming University of Science and Technology, Kunming, Yunnan, China;; b Department of Nephrology, Xiangya Hospital, Central South University, Changsha, Hunan, China

**Keywords:** Acteoside, IgA nephropathy, Th22 Cell, inflammation, chemotaxis

## Abstract

The existing therapies of IgA nephropathy are unsatisfying. Acteoside, the main component of *Rehmannia glutinosa* with anti-inflammatory and anti-immune effects, can improve urinary protein excretion and immune disorder. Th22 cell is involved in IgA nephropathy progression. This study was determined to explore the effect of acteoside on mesangial injury underlying Th22 cell disorder in IgA nephropathy. Serum Th22 cells and urine total protein of patients with IgA nephropathy were measured before and after six months treatment of *Rehmannia glutinosa* acteoside or valsartan. Chemotactic assay and co-culture assay were performed to investigate the effect of acteoside on Th22 cell chemotaxis and differentiation. The expression of CCL20, CCL22 and CCL27 were analyzed. To explore the effect of acteoside on mesangial cell injury induced by inflammation, IL-1, IL-6, TNF-α and TGF-β1 were tested. Results showed that the proteinuria and Th22 lymphocytosis of patients with IgA nephropathy significantly improved after combination treatment of *Rehmannia glutinosa* acteoside and valsartan, compared with valsartan monotherapy. *In vitro* study further demonstrated that acteoside inhibit Th22 cell chemotaxis by suppressing the production of Th22 cell attractive chemokines, i.e., CCL20, CCL22 and CCL27. In addition, acteoside inhibited the Th22 cell proliferation. Co-culture assay proved that acteoside could relieve the overexpression of pro-inflammatory cytokines, and prevent the synthesis of TGF-β1. TGF-β1 level in mesangial cells was positively correlated with the Th22 cell. This research demonstrated that acteoside can alleviate mesangial cell inflammatory injury by modulating Th22 lymphocytes chemotaxis and proliferation.

## 1. Introduction

IgA nephropathy (IgAN) is the most common cause of end-stage renal disease. About 30–40% of patients with IgAN may develop into ESRD in 20–30 years [1]. Recently published Kidney Disease/Improving Global Outcomes (KDIGO) guideline makes it clear that the effectiveness of the existing therapies is limited in targeted treatments, and therapy which is more specific to the pathogenic process in IgAN is still needed [2].

*Rehmannia glutinosa* is a traditional medicine, which is widely used in Chinese, Russian and Japanese medicine for its anti-infection, anti-inflammation and immune-regulation abilities. Acteoside (ACT), the main component of *Rehmannia glutinosa*, has a wide range of effects, including regulation of immune and inflammatory responses [3]. Previous researches demonstrated that acteoside could improve urinary protein excretion and decrease the incidence of mesangial expansion [4,5]. This makes acteoside a possible therapy of IgAN.

T helper type 22 (Th22) lymphocyte is a newly identified CD4^+^ T helper (Th) lymphocyte subset characterized by the expression of CCR4, CCR6 and CCR10. Th22 cell disorder is involved in the immunopathologic and inflammatory processes in IgAN [6,7]. The adverse impact of Th22 cell overexpression in IgAN partly attributes to CCL20, CCL22 and CCL27 activities [8]. Thus, this research is determined to explore the underlying immune and inflammatory regulation effect of acteoside on mesangial injury in IgA nephropathy based on Th22 cell disorder.

## 2. Materials and methods

### 2.1. Ethics statement

This study protocol was approved by the medical ethics committee of the Xiangya Hospital of Central South University for Human Studies (approval number 201403270), and all subjects signed informed consents.

### 2.2. Subjects

Sixteen healthy controls and 31 adults from Xiangya Hospital during 2015–2016, aged between 18–64 years with biopsy confirmed IgAN, were included. Patients were excluded if they had acute infection, glucocorticoid or immunosuppressant treatments, or other complications. Demographic, clinical and biochemical characteristics of patients with IgAN were shown in Table 1.

**Table 1.** Demographic, clinical, and biochemical characteristics, and pathology of subjects.

	Healthy control (*N* = 16)	Acteoside + valsartan group (*N* = 21)	Valsartan group (*N* = 10)
Male (*n*, *n*%)	5 (31.25%)	10 (47.61%)	5 (50%)
Age (years)	26.69 ± 1.35	30.25 ± 13.41	46 ± 16.47
Serum albumin (g/L)	43.26 ± 3.21	38.70 ± 4.69*	39.09 ± 7.11*
Blood urea nitrogen (mmol/L)	4.78 ± 0.84	5.87 ± 2.88*	6.78 ± 5.79*#
Serum creatinine (umol/L)	81.38 ± 13.74	101.38 ± 72.27*	97.27 ± 21.53*#
Uric acid (umol/L)	312.56 ± 55.82	337.15 ± 97.88*	319.16 ± 62.34
Pathological Lesions (*n* (*n*%)			
Mesangial hypercellularity (M1)		21 (100%)	10 (100%)
Endocapillary hypercellularity (E1)		6 (28.57%)	8 (57.14%)
Segmental glomerulosclerosis (S1)		1 (4.761%)	13 (92.86%)
Tubular atrophy/interstitial fibrosis (T1)		1 (4.761%)	3 (21.43%)

* *p* < .01 compared with healthy controls; #*p* < .01 compared with acteoside + valsartan group.

### 2.3. Acteoside attenuates proteinuria and Th22 lymphocytosis in IgAN

Eleven patients with IgAN were treated with valsartan (80–160 mg/d; Beijing Novartis Pharma Ltd, Beijing, China). Twenty-one IgAN patients were treated with *Rehmannia glutinosa* acteoside (0.8 g/d; Sichuan Medo Pharmaceutical Stock Co., Ltd, Sichuan, China) combined with valsartan (80–160 mg/d; Beijing Novartis Pharma Ltd, China). Twenty-four hour urinary protein were quantitatively analyzed. Peripheral blood Th22 lymphocytes were analyzed by flow cytometry as CD3^+^CD4^+^IL-17^−^INF-γ^−^IL-22^+^ lymphocytes. CD3 (PerCP-Cyanine5.5; eBioscience, Vienna, Austria), CD4 (FITC; eBioscience), CCR4 (APC; eBioscience), CCR6 (PE-Cyanine7; eBioscience), and CCR10 (PE; BioLegend, CA, USA) on T-cells isolated from the blood of patients with IgAN, and IFN-γ (APC; eBioscience), IL-22 (PE; eBioscience), IL-17 A (PE-Cyanine7; eBioscience), and Ki67 (Alexa 700 MAB; BD Biosciences, CA, USA) were stained using the fixation/permeabilization concentrate kit (BD Biosciences) according to the manufacturer’s instructions and analyzed by flow cytometry.

### 2.4. Acteoside reduces theTh22 cell chemotaxis assays

CD4^+^ T lymphocytes from patients with IgAN were isolated and purified using a CD4^+^ T cell isolation kit according to the manufacturer’s instructions (Miltenyi Biotec, Bergisch Gladbach, Germany). Purified CD4^+^ T lymphocytes from each IgAN patient were separated into three groups, i.e., control group, HMC group and acteoside group. These CD4^+^ T lymphocytes were added into the upper chambers of a 24-well transwell plate (Corning Costar, New York, USA) in RPMI-1640 medium with 0.5% FBS in a final volume of 100 μL. The lower chambers of transwell plate were filled with 600 μL of supernatant of cultured human mesangial cells (HMCs) (purchased from Central South University Advanced Research Center, Hunan, China). Acteoside (40 mM; purchased from Sichuan Medo Pharmaceutical Stock Co., Ltd, Sichuan, China) was used to interfere the Th22 cell chemotaxis. The transwell chambers were placed at 37 °C in 5% CO_2_ for 5 h. Th22 cells migrated into the lower chamber were analyzed by flow cytometry, and assigned a chemotaxis index (chemotaxis index = number of migrated Th22 cells in each experiment group ÷ number of migrated Th22 cells in response to medium alone). The concentrations of CCL20, CCL22 and CCL27 were quantified by using enzyme-linked immunosorbent assay (ELISA) kits (R&D, MN, USA) according to the manufacturer’s instructions.

### 2.5. Acteoside inhibits Th22 cell differentiation *in vitro*

CD4^+^ T lymphocytes were treated with 20 mM to 100 mM acteoside in dose-dependent effect assay. CD4^+^ T lymphocytes were treated with 40 mM acteoside for 24 h to 168 h in time-dependent effect assay. IL-2 (2 ng/mL), anti-CD3 and anti-CD28 mAbs (1 μg/mL each) were used to ensure the survival of T lymphocyte. The exogenous cytokines used to modulate cell differentiation were IL-1β (20 ng/mL), IL-6 (100 mg/mL), TNF-α (50 ng/mL), as well as acteoside (40 mM) alone or in combination. All the reagents used for cell differentiation assays were purchased from PeroTech, Rocky Hill, CT, USA.

### 2.6. Acteoside relieves Th22 cell overexpression related HMCs inflammatory injury

Experiments were independent biological repeats. CD4^+^ T lymphocytes of patients with IgAN were not pooled. CD4^+^ T lymphocytes drawn from one patient with IgAN at a single time point were divided into different treatment groups in one repeat experiment. Purified CD4^+^ T lymphocytes from IgAN patient were separated, co-cultured with HMCs in T + HMC group and acteoside group at a ratio of 5:1 with RPMI-1640 supplemented and 12% fetal bovine serum (FBS) for 5 days. Acteoside (40 mM) were applied into acteoside group. Th22 cells were harvested and analyzed by flow cytometry. IL-1, TNF-α and IL-6 levels in the supernatant were quantified by using ELISA kits (R&D, USA) according to the manufacturer’s instructions. The immunoreactivity was determined by using an ELISA reader at 450 nm.

### 2.7. Acteoside alleviates the synthesis of TGF-β1

Transforming growth factor beta 1 (TGF-β1) in HK2 cells and HMCs was analyzed by Western blot. Adherent cells in the co-culture system were harvested, lysed for 10 min on ice with 80 μL of RIPA buffer (Well Biology, Peking, China), and centrifuged (12,000 ×*g* for 15 min). Lysate samples (50 g) were boiled in sample buffer for 5 min and separated on 10% SDS-PAGE, followed by transfer onto a nitrocellulose membrane (Millipore, MA, USA). The membrane was incubated with a monoclonal rabbit anti-human TGF-β1 antibody (Proteintech, Chicago, IL, USA) and reacted with an anti-mouse IgG-HRP antibody (Proteintech). TGF-β1 levels were normalized to β-actin levels and quantified using ECL chemiluminescence system (Thermo Scientific Pierce, Shanghai, China). Films were scanned and images were analyzed using Quantity One 4.62 software (Bio-rad, CA, USA).

### 2.8. Statistical analysis

Data were expressed as mean ± standard deviation (SD) or median with minimum and maximum value. Data comparisons were performed using Kruskal–Wallis one-way analysis or Mann–Whitney *U*-test of variable for ranking. Comparison of urinary protein excretion and Th22 cell expression before and after drug therapy was performed by using repeated measurement ANOVA. Variables in chemotaxis assay and co-culture assay were compared using Students’s *t*-test or the Wilcoxon signed-rank test. The correlations among variables were determined by calculating the Spearman rank correlation coefficients. *p* < .05 was set as the statistical significance. The exact *p* values were expressed as *p* < .001 if the *p* values was less than 1 × 1.0^−3^. Statistical analyses were performed by using SPSS 19.0 software package (International Business Machines Corporation, Chicago, IL, USA).

## 3. Results

### 3.1. Rehmannia glutinosa acteoside relieves proteinuria and Th22 lymphocytosis in IgAN

**Figure 1.**
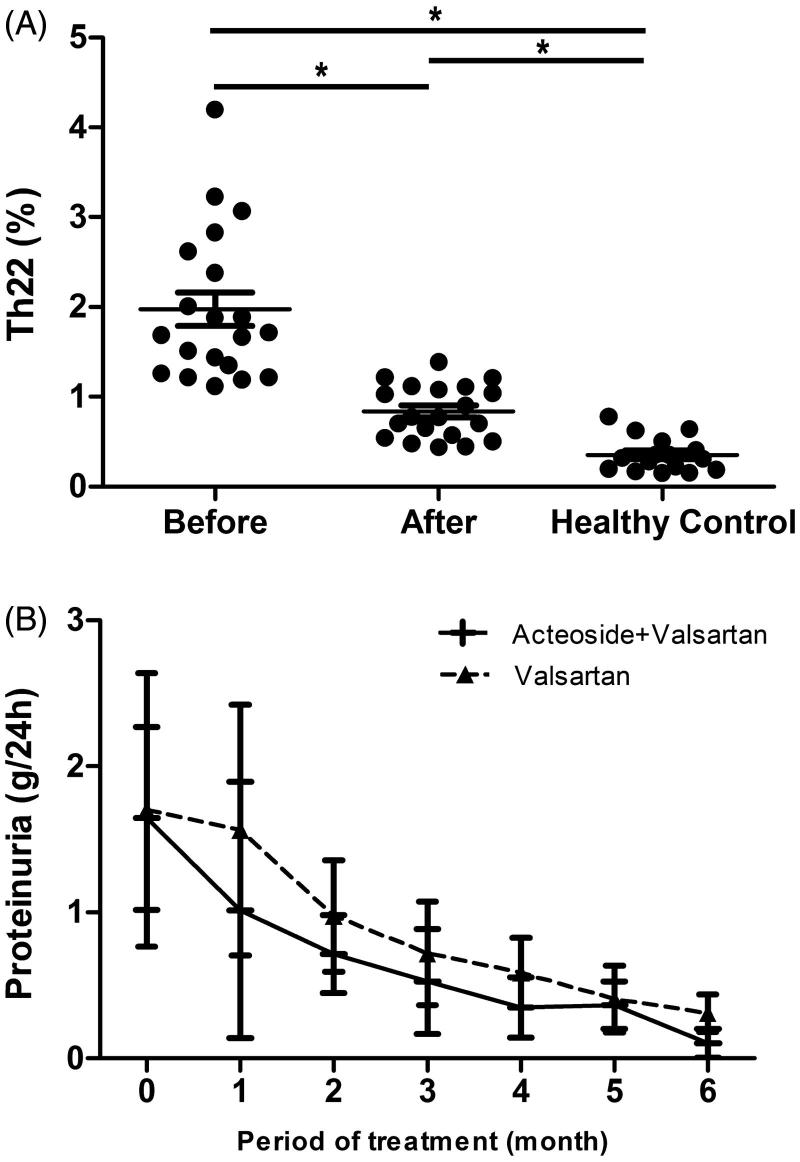
ACT attenuates Th22 cell disorder and proteinuria in IgAN. Patients with IgAN were treated with *Rehmannia glutinosa* acteoside (0.8 g/d) and valsartan (0.08–0.16 g/d) for 8 months. (A) Results of the Th22 cell amounts in IgAN patients before and after 6 months *Rehmannia glutinosa* acteoside and valsartan therapy comparing with healthy controls. * represents *p* < .01. (B) Results of 24 h urinary protein quantity analysis for IgAN patients. There is no difference in total urinary protein between valsartan group and valsartan + acteoside group before drug therapy (*p* = .853). Six months later, the post treatment total urinary protein of acteoside + valsartan group significantly decreases comparing with valsartan group (*p* = .015).

As illustrated in Figure 1(A), proportion of peripheral blood Th22 cells in IgAN patients (1.08 ± 0.83%) increased compared with healthy controls (0.35 ± 0.19%; *p* < .001). After 6 months combination therapy of *Rehmannia glutinosa* acteoside and valsartan (acteoside *+* valsartan group), Th22 cell proportion decreased (0.85 ± 0.29%; *p* < .001), along with a remission in urinary protein excretion (1.64 ± 0.62 g/d vs. 0.10 ± 0.09 g/d) (Figure 1(B)).

Comparison of urinary protein excretion between valsartan group and acteoside *+* valsartan group before therapy did not show any significant difference (1.70 ± 0.94 g/d vs. 1.64 ± 0.63 g/d; *p* = .853). However, urinary protein excretion of acteoside + valsartan group (0.11 ± 0.09 g/d) significant decreased after 6 months treatment, compared with valsartan group (0.31 ± 0.13 g/d; *p* = .015).

### 3.2. Acteoside inhibits Th22 cell chemotaxis

Th22 cells lymphocytosis is closely correlated with the aggravation of IgAN [8]. The accumulation of Th22 cells may attribute to lymphocyte infiltration and proliferation. Lymphocyte migration that responded to chemokines activities is the key to Th22 cell infiltration. Chemotaxis assay confirmed that acteoside inhibited Th22 cell migration. Results showed that the Th22 cell chemotactic index of T + HMC group markedly increased compared with controls (10.31 ± 0.52 vs. 3.19 ± 0.0.31; *p* < .001). By contrast, Th22 cells chemotactic index of acteoside group decreased (3.27 ± 0.0.31; *p* < .001).

The Th22 cell chemotaxis response is regulation by the activities of Th22 cell attractive chemokines, i.e., CCL20, CCL22 and CCL27. We further testified the effect of acteoside on the production of CCL20, CCL22 and CCL27. As expected, an overexpression of CCL20 (96.70 ± 4.20 pg/mL), CCL22 (907.19 ± 8.04 pg/mL) and CCL27 (404.70 ± 12.59 pg/mL) were observed in T + HMC co-culture group compared with HMC group (36.20 ± 2.81 pg/mL, 36.20 ± 2.81 pg/mL and 100.13 ± 9.85 pg/mL, *p* < .001, respectively), while acteoside significantly inhibited the secretion of CCL20, CCL22 and CCL27 (46.53 ± 2.07 pg/mL, 553.35 ± 13.63 pg/mL, and 321.4 ± 11.22 pg/mL; *p* < .001, respectively).

Consisted with the reducing production of CCL22, CCL20 and CCL27, the expression of chemokine receptors, i.e., CCR4, CCR6 and CCR10, on recruited Th22 cells decreased in acteoside group, compared with HMC group (CCR4: 6.32 ± 0.95% vs. 4.76 ± 0.98%, CCR6: 1.28 ± 0.08% vs. 0.23 ± 0.07%, CCR10: 21.76 ± 1.27% vs. 8.67 ± 1.41%; *p* < .001).

### 3.3. Acteoside inhibits Th22 cell proliferation

**Figure 2.**
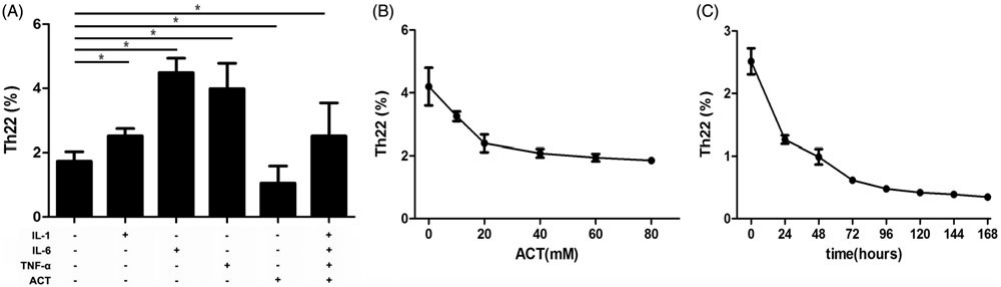
ACT inhibits Th22 cell differentiation ***in vitro***. (A) The proportion of Th22 cells, which are differentiated from purified CD4^+^ T lymphocytes from IgAN patients by stimulation with different combinations of IL-1, IL-6, TNF-α and ACT for 7 days. Media contains anti-CD3, anti-CD28 and IL-2 to ensure T-cell activation. *represents *p* < .01. (B) Dose-related effects of ACT on Th22 cell differentiation. Purified CD4^+^ T lymphocytes isolated from IgAN patients are treated with different doses of ACT for 96 h. (C) Time-dependent effects of ACT on Th22 cells differentiation. Purified CD4^+^ T lymphocytes isolated from IgAN patients are treated with 40 mM ACT for 24 to 168 h.

Th22 cell differentiation and proliferation also contribute to the Th22 cell accumulation. Th22 cell proliferation in acteoside group was significantly down-regulated compared with control group (1.06 ± 0.29% vs. 1.73 ± 0.29%; *p* < .001). Moreover, acteoside inhibited the Th22 cell proliferation in a dose and time-depend manner (Figures 2(A,B).

IL-1, IL-6 and TNF-α are well-known accelerators of Th22 cell differentiation. As shown in Figure 2(C), treatment of IL-1, IL-6 and TNF-α promoted Th22 cell proliferation (2.51 ± 0.22%, 4.49 ± 0.45%, 3.98 ± 1.08%). However, the Th22 cell proliferation in groups treated with acteoside (1.06 ± 0.53%, *p* < .001) or a combination of IL-1, IL-6, TNF-α and acteoside (3.51 ± 1.03%) decreased.

### 3.4. Acteoside relieves Th22 cells related inflammation response and HMCs injury

The Th22 cell percentage decreased in co-culture systems of acteoside group (2.51 ± 4.54%) compared with T + HMC group (4.54 ± 0.45%, *p* < .001).

Accordingly, the levels of pro-inflammatory cytokines, i.e., IL-1, IL-6 and TNF-α, were up-regulated in T + HMC group, while acteoside treatment inhibited their synthesis (IL-1: 128.30 ± 5.91 vs. 173.44 ± 1.60 pg/mL, IL-6: 53.35 ± 1.98 vs. 65.87 ± 9.61 ng/mL, TNF-α: 231.51 ± 2.48 vs. 301.58 ± 10.71 pg/mL; *p <* .001, respectively). The Th22 cell amount was positively correlated with the IL-1, IL-6 and TNF-α levels (*r* were 0.853, 0.829 and 0.747, respectively; *p* < .001).

**Figure 3.**
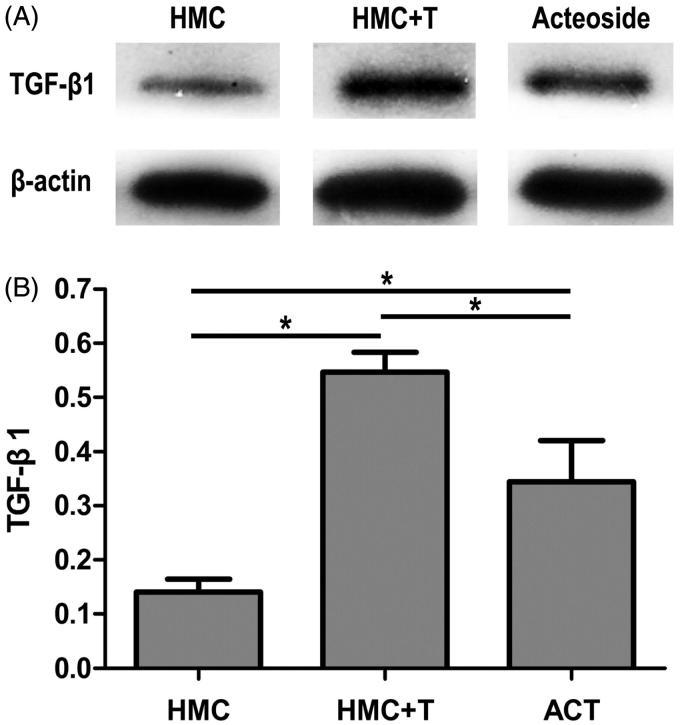
ACT suppresses the synthesis of TGF-β1. CD4^+^ T lymphocytes of patients with IgAN are co-cultured with HMCs for 5 days. ACT (40 mM) is applied to regulate inflammatory response. (A) TGF-β1 protein levels in HMCs are analyzed by Western blot. (B) Bars represent the concentration of TGF-β1 in HMCs (*n* = 5). * represents *p* < .01.

Though the up-regulation of pro-inflammatory cytokines indicates the exaggeration of inflammatory response [9–11], a change in biomarker of mesangial injury may be more intuitively in showing us the impact of inflammation. Thus, we detected the content of TGF-β1 in mesangial cells. As illustrated in Figure 3, Western blot analysis demonstrated a markedly increase in TGF-β1 in T + HMC group (0.55 ± 0.04) comparing with HMC group (0.15 ± 0.01; *p* < .001), while TGF-β1 in HMCs of acteoside group decreased significantly (0.34 ± 0.07, *p* = .003). This mesangial cell protective effect of acteoside was positively correlated with the changes of Th22 cells, IL-1, IL-6 and TNF-α (*r* were 0.877, 0.865, 0.883 and 0.906, respectively; *p* < .001).

## 4. Discussion

IgAN is the most common primary glomerulonephritis. Patient with IgAN, even if treated with standard therapy, still face an irreversible exacerbation of disease and a loss of renal function over time [2,12]. There is an urgent need to search for more effective therapies. Acteoside is the main component of *Rehmannia glutinosa* with anti-bacterial, antioxidant, anti-inflammation, and immunoregulation activities. These make acteoside a possible therapy for IgAN. Hong et al. have proved that urinary protein levels were more effectively controlled in patients with chronic nephritis treated with *Rehmannia glutinosa* acteoside and irbesartan compared with irbesartan [13], but the effect of acteoside in IgAN is unknown.

We found that *Rehmannia glutinosa* acteoside could significantly improve the proteinuria and Th22 cell overexpression of patients with IgAN. This result was consistent with Sadakane’s research of nephritis mice, which included an IgAN subgroup and showed that the administration of acteoside improved renal function [14,15]. But they did further explore the relevant mechanism. According to Wei Y et al. research, acteoside could relieve inflammation response by adjusting Th cell disorder [16]. It is possible that acteoside may improve renal damage by regulating Th cell disorder-related inflammation response in IgAN.

Th22 lymphocytosis participates to IgAN progression [17]. Xiao C et al. demonstrated that the increasing Th22 cell in mice with IgAN was positively correlated with renal lesions [8]. Thus, therapies that can ameliorate Th22 cell migration and proliferation may improve renal injury in IgAN. Our result suggested that acteoside could alleviate proteinuria and Th22 lymphocytosis in IgAN, indicating that acteoside may relieve renal injury by regulating Th22 cell disorder.

The accumulation of Th22 cell may attribute to either cell infiltration or cell proliferation. In this research, we demonstrated that acteoside could directly inhibit the Th22 cell chemotaxis, which may induce an accumulation of Th22 cells in kidney. Grigore et al. demonstrated that acteoside could inhibit leukocytes infiltration and improve renal inflammation [18,19], but the mechanism was not explained. We confirmed that the immunoregulation and renal-protective effect of acteoside based on Th22 cell regulation was partly relying on the regulation of CC chemokines expression.

Chemokines is essential to lymphocyte migration. Th22 cell chemotaxis is in response to the effects of CCL22, CCL20 and CCL27, which are the members of CC chemokines. The collaboration between these chemokines and their corresponding receptors expressed on Th22 cells may contribute to lymphocyte disorder in IgAN [6]. Researches proved that infection and galactose-deficient IgA deposition may induce mesangial cell to secrete chemokines, thus resulting in mesangial proliferation and podocyte injury [6,20,21]. We found that the renal-protective effect of acteoside was underlying and attributed to the effect of CCL20, CCL22 and CCL27. We demonstrated that acteoside could prevent mesangial cell from expressing CCL20, CCL22 and CCL27, thus alleviating Th22 cell migration. Though no evidence has confirmed the effectiveness of acteoside to regulate the expression of chemokines in IgAN, Motojima H et al. did provide a proof that acteoside could alleviate inflammatory response by down-regulating the expression of CCL1, CCL2, CCL3 and CCL4 in allergy [22].

The effectiveness of acteoside also relies on regulating lymphocyte proliferation. We demonstrated that acteoside could suppress Th22 cell differentiation in a time and dose-dependent manner. Yoou MS et al. declared that acteoside attenuates inflammatory mast cell proliferation via down-regulating MDM2 [23].

In view of the adverse effect of Th22 cell in IgAN, the activities of acteoside to alleviate Th22 cells chemotaxis and proliferation make it possible to improve kidney damage. Saegusa Y et al. suggested that acteoside had a mesangial protection activity [15]. Our results indicated that acteoside significantly improved Th22 cell disorder and inhibited mesangial cells to secrete pro-inflammatory cytokines, i.e., IL-1, IL-6 and TNF-α, thus alleviating the inflammatory response.

Since chronic inflammation is a common hallmark of renal fibrosis in IgAN [10,11], we further investigated the effect of acteoside on mesangial cell fibrosis by investigating the synthesis of TGF-β1 in mesangial cells. Data suggested that acteoside significantly inhibit the TGF-β1 production in mesangial cells. This decrease in TGF-β1 was correlated with the levels of Th22 cells and pro-inflammatory cytokines. Brembilla et al. showed that Th22 cells could capacitate a pro-inflammatory phenotype fibroblast in response to TNF [24]. Research of Sziksz E et al. proved that the Th22 cell attractive towards chemokine CCL20 are profibrotic [25]. Weathington confirmed that IL-22, the main effect molecule of Th22 cell, can bind to the IL-22RA receptor, activating JAKI–STAT1 and STAT3 pathways, regulating Akt, ERK, JNK and p38 signal, thus participating in the regulation of renal fibrosis [26]. These demonstrated that acteoside can alleviate mesangial cell inflammatory injury by modulating Th22 lymphocytes disorder.

## 5. Conclusions

This study demonstrated that Th22 cell disorder and the relevant inflammatory response attributed to the progress of IgAN. Acteoside could serve as an alternative therapeutic for IgAN by remitting Th22 cell related-inflammatory injury and immune disorder.
